# FcRγ Controls the Fas-Dependent Regulatory Function of Lymphoproliferative Double Negative T Cells

**DOI:** 10.1371/journal.pone.0065253

**Published:** 2013-06-06

**Authors:** Stephen C. Juvet, Christopher W. Thomson, Edward Y. Kim, Mei Han, Li Zhang

**Affiliations:** 1 Institute of Medical Science, University of Toronto, Toronto, Ontario, Canada; 2 Division of Respirology and Toronto Lung Transplant Program, Department of Medicine, University Health Network, University of Toronto, Toronto, Ontario, Canada; 3 Clinician-Scientist Training Program, Department of Medicine, University of Toronto, Toronto, Ontario, Canada; 4 Department of Laboratory Medicine and Pathobiology, University of Toronto, Toronto, Ontario, Canada; 5 Department of Immunology, University of Toronto, Toronto, Ontario, Canada; 6 Toronto General Research Institute, University Health Network, Toronto, Ontario, Canada; Beth Israel Deaconess Medical Center, Harvard Medical School, United States of America

## Abstract

Patients with autoimmune lymphoproliferative syndrome (ALPS) and lymphoproliferation (LPR) mice are deficient in Fas, and accumulate large numbers of αβ-TCR^+^, CD4^−^, CD8^−^ double negative (DN) T cells. The function of these DN T cells remains largely unknown. The common γ subunit of the activating Fc receptors, FcRγ, plays an important role in mediating innate immune responses. We have shown previously that a significant proportion of DN T cells express FcRγ, and that this molecule is required for TCR transgenic DN T cells to suppress allogeneic immune responses. Whether FcRγ plays a critical role in LPR DN T cell-mediated suppression of immune responses to auto and allo-antigens is not known. Here, we demonstrated that FcRγ^+^, but not FcRγ^−^ LPR DN T cells could suppress Fas^+^ CD4^+^ and CD8^+^ T cell proliferation *in vitro* and attenuated CD4^+^ T cell-mediated graft-versus host disease. Although FcRγ expression did not allow LPR DN T cells to inhibit the expansion of Fas-deficient cells within the LPR context, adoptive transfer of FcRγ^+^, but not FcRγ^−^, DN T cells inhibited lymphoproliferation in generalized lymphoproliferative disease (GLD) mice. Furthermore, FcRγ acted in a cell-intrinsic fashion to limit DN T cell accumulation by increasing the rate of apoptosis in proliferated cells. These results indicate that FcRγ can confer Fas-dependent regulatory properties on LPR DN T cells, and suggest that FcRγ may be a novel marker for functional DN Tregs.

## Introduction

Fas-deficient lymphoproliferation (LPR) and FasL-deficient generalized lymphoproliferative disease (GLD) mice, and humans with autoimmune lymphoproliferative syndrome (ALPS) develop marked lymphoproliferation. They also exhibit lupus-like autoimmunity that is largely dependent on B cells [1] and CD4^+^ T cells [2], [3]. Furthermore, they accumulate large numbers of TCRαβ^+^CD4^−^CD8^−^ double negative (DN) T cells.

The function of DN T cells in LPR mice is not clear. Two published studies have illustrated that these cells can exert regulatory function outside the LPR context, toward T cells responding to alloantigens [4], [5]. Within Fas-deficient mice and humans, however, evidence suggests that DN T cells contribute to disease, either by promoting further lymphocyte accumulation [6] or by promoting autoimmune tissue injury [7], [8]. It has long been recognized, however, that the DN T cell compartment of LPR mice is heterogeneous and may contain cells with differing functional properties [9]. Hence, the identification of molecules that segregate with specific DN T cell functions is of interest.

The common γ subunit of the activating Fc receptors, FcRγ, is a signal transducing adaptor protein that plays a central role in linking the specificity of immunoglobulins with the effector functions of the innate immune system [10]. It is critical to NK cell-mediated antibody-dependent cell-mediated cytotoxicity (ADCC), phagocytosis by macrophages, and mast cell responses to IgE crosslinking [11]. FcRγ is also highly homologous to the key T cell receptor (TCR) signaling molecule, CD3ζ [12] and can substitute for it during T cell development [13]. FcRγ has been found in the TCR complexes of certain intraepithelial T cells [14], [15], the CD4^+^ and CD8^+^ T cells of lupus patients [16], and human effector CD4^+^ T cells [17]. FcRγ-containing TCR complexes have been associated with some differences in signal transduction compared with CD3ζ-containing ones [18], [19]. However, the specific immunological consequences of FcRγ expression in T cells have not been clearly demonstrated.

DN T cells bearing the L^d^-specific 2C transgenic TCR have been shown to be capable of inhibiting allogeneic immune responses mediated by L^d^-specific 2C CD8^+^ T cells [20], including the rejection of skin [20], [21] and cardiac [22] allografts, and GVHD [23]. We observed that among 1099 genes differentially expressed between regulatory and non-regulatory DN T cell clones, FcRγ was the most highly upregulated in the regulatory ones [24]. We subsequently showed that FcRγ inclusion in the TCR complex of 2C DN T cells was required for their regulatory function [25]. Whether FcRγ expression is required for non-transgenic DN T cells to perform regulatory functions is not known.

To determine whether FcRγ might play a role in the regulatory function of LPR DN T cells, we generated LPR FcRγ^−/−^ mice on the C57BL/6 background and observed that they exhibited increased T cell accumulation and early mortality compared with LPR FcRγ^+/+^ mice [26]. This observation led us to hypothesize that FcRγ expression by LPR DN T cells might participate in controlling lymphocyte accumulation in LPR mice. Here, we show that FcRγ expression is critical for LPR DN T cells to exert regulatory function toward Fas-sufficient CD4^+^ and CD8^+^ T cells responding to auto- and allo-antigens *in vitro and in vivo*. In addition, FcRγ cell-intrinsically determined the rate of DN T cell accumulation in LPR mice. Hence, our data reveal that FcRγ plays an important role in controlling the function and survival of LPR DN T cells.

## Results

### A Subset of FcRγ^+^ LPR DN T Cells Exhibits an Effector-memory Phenotype

**Figure 1:**
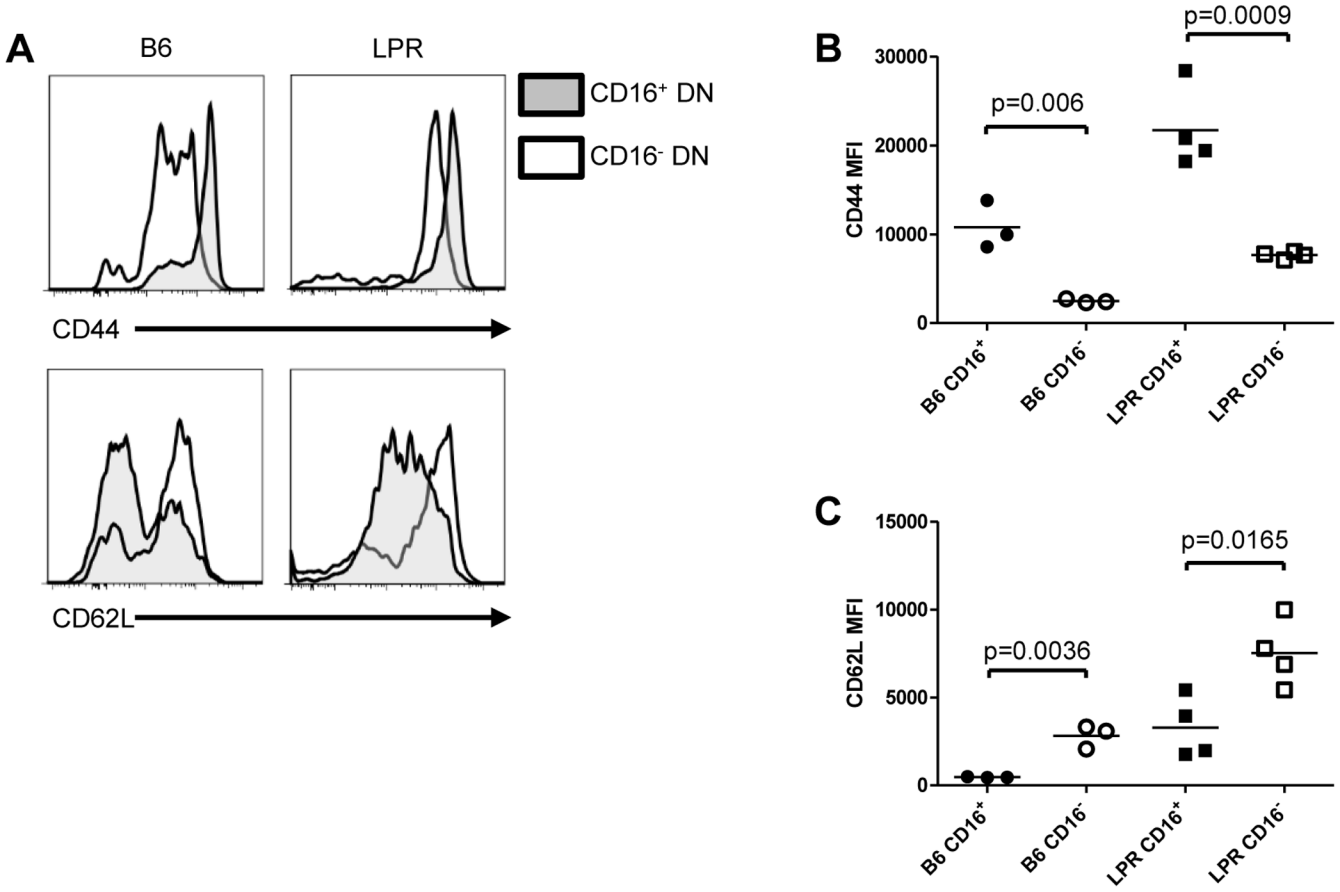
FcRγ-expressing DN T cells are a distinct effector-memory subset in both B6 and LPR mice. **A.** Freshly isolated B6 (n = 3, left column) and LPR (n = 4, right column) splenocytes were stained for TCRβ, CD4, CD8, NK1.1, CD16/32, CD44, and CD62L expression and examined by flow cytometry. Within the DN T cell gate (TCRβ^+^, CD4^−^, CD8^−^, NK1.1^−^), expression of CD44 (top row) and CD62L (bottom row) in the CD16^+^ (shaded) and CD16^−^ (unshaded) subsets was plotted. **B.** Median fluorescence intensity (MFI) of CD44 staining in CD16^+^ and CD16^−^ DN T cells for all 7 mice is shown. Unpaired t tests p = 0.006 (CD16^+^ vs. CD16^−^ B6 DN T cells) and p = 0.0009 (CD16^+^ vs. CD16^−^ LPR DN T cells). **C.** MFI of CD62L staining in CD16^+^ and CD16^−^ DN T cells for all 7 mice is shown. Unpaired t tests p = 0.0036 (CD16^+^ vs. CD16^−^ B6 DN T cells) and p = 0.0165 (CD16^+^ vs. CD16^−^ LPR DN T cells).

FcRγ is well-known to be expressed in myeloid cells, NK cells and B cells but its expression in T cells is less common. We recently observed that in contrast to CD4^+^ or CD8^+^ T cells, a significant proportion of DN T cells co-expresses FcRγ and surface Fcγ receptor IIIA (CD16) [26]. Since little is known about FcRγ^+^ DN T cells, we further characterized this population of cells. To examine their expression of T cell activation and memory markers, splenocytes from B6 and LPR mice were stained for expression of TCRβ, CD4, CD8, NK1.1, CD16/32 and CD44, CD62L, or CD25. As shown in **Fig. 1A–C**, CD16^+^ DN T cells (TCRβ^+^, CD4^−^, CD8^−^, NK1.1^−^) exhibited higher levels of CD44 expression, and lower levels of CD62L expression, in comparison with CD16^−^ DN T cells within the same mice. Although LPR DN T cells are known to have an activated phenotype with high levels of CD44 expression [9], [27], we observed even higher levels of CD44 expression on the CD16^+^ subset (**Fig. 1A** **, top right panel** and **Fig. 1B**). A higher level of CD25 expression was also seen in the CD16-expressing subset (**data not shown**). Hence in both normal B6 mice and Fas-deficient LPR mice, an FcRγ-expressing subset of DN T cells displays an effector-memory phenotype.

### FcRγ Restrains DN T Cell Expansion in vitro and in vivo

**Figure 2:**
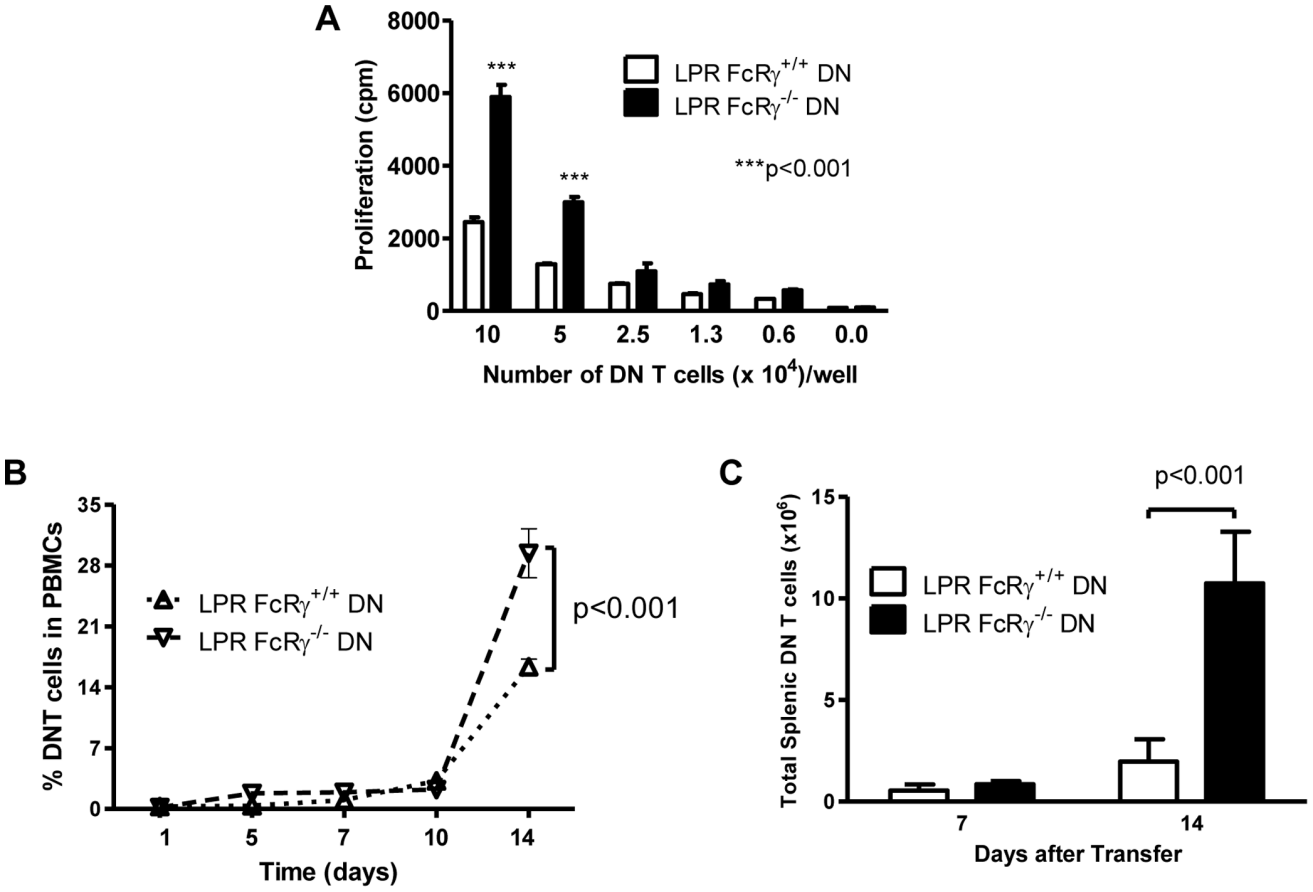
FcRγ deficiency results in an increased accumulation of DN T cells. **A.** B6.LPR.FcRγ^+/+^ and B6.LPR.FcRγ^−/−^ mice were given 4×10^7^ CB6F1 splenocytes intravenously. After 7 days, varying numbers of DN T cells were purified and incubated for a further 3 days with irradiated CB6F1 splenocytes (10^5^/well), after which 1 µCi ^3^H-thymidine was added to each culture. Thymidine uptake, reflecting live proliferated cell number, was determined by scintillation counting. Two-way ANOVA p<0.0001; **Bonferroni post tests p<0.001. **B.** B6.SCID mice (FcRγ^+/+^) received 10^7^ B6.LPR.FcRγ^+/+^ (n = 3, upright triangles) or B6.LPR.FcRγ^−/−^ (n = 3, inverted triangles) DN T cells. On days 1, 5, 7, 10, and 14 blood samples were obtained and peripheral blood mononuclear cells (PBMCs) were stained for TCRβ, CD4, CD8, and NK1.1 expression. The percentage of DN T cells in the PBMC compartment was then determined by flow cytometry. Two-way repeated measures ANOVA p = 0.0279 for the effect of FcRγ genotype; Bonferroni post test p<0.001 at day 14. **C.** At day 7 and day 14, the splenocytes of the DN T cell recipients were counted and stained for TCRβ, CD4, CD8, and NK1.1 and examined by flow cytometry. The number of splenic DN T cells in each type of recipient was then determined. Two-way ANOVA p = 0.0005 for the effect of FcRγ genotype; Bonferroni post test p<0.001 at day 14.

Since LPR FcRγ^−/−^ mice exhibit a much greater accumulation of DN T cells than LPR FcRγ^+/+^ mice [26], we hypothesized that FcRγ expression might inhibit DN T cell proliferation. To test this hypothesis, LPR FcRγ^+/+^ or LPR FcRγ^−/−^ DN T cells were preactivated *in vivo* by infusing LPR FcRγ^+/+^ and LPR FcRγ^−/−^ mice with allogeneic CB6F1 splenocytes. The purified DN T cells were then cultured in varying ratios with irradiated CB6F1 splenocytes and IL-2. Proliferation of DN T cells was measured by ^3^H-thymidine incorporation. As shown in **Fig.** **2A**, LPR FcRγ^−/−^ DN T cells proliferated significantly greater than that of LPR FcRγ^+/+^ DN T cells. These data suggest that FcRγ expression in LPR DN T cells decreases their propensity to proliferate in response to alloantigen-stimulation *in vitro.*

Next, we assessed whether FcRγ expression might contribute to LPR DN T cell expansion *in vivo*. We adoptively transferred LPR FcRγ^+/+^ or LPR FcRγ^−/−^ DN T cells into syngeneic B6.SCID mice (FcRγ^+/+^) and assessed their expansion in the peripheral blood at days 1, 5, 7, 10, and 14. As shown in **Fig. 2B**, both types of DN T cells accounted for a very small fraction of PBMCs until day 14, when preferential expansion of LPR FcRγ^−/−^ DN T cells was evident (two-way repeated measures ANOVA p = 0.0279 for the effect of FcRγ). Similarly, when splenic DN T cell counts were determined at day 7, a similar number were found in recipients of both types of DN T cell; at day 14, however, the number of DN T cells recovered from recipients of LPR FcRγ^−/−^ DN T cells was approximately 5 times greater (**Fig. 2C**, two-way ANOVA p = 0.0005 for the effect of FcRγ expression by transferred DN T cells). Together, these data suggest that FcRγ expression in DN T cells limits their proliferation *in vitro* and in a lymphopenic environment.

### FcRγ-expressing DN T Cells are Lost with Increasing Lymphocyte Accumulation

**Figure 3:**
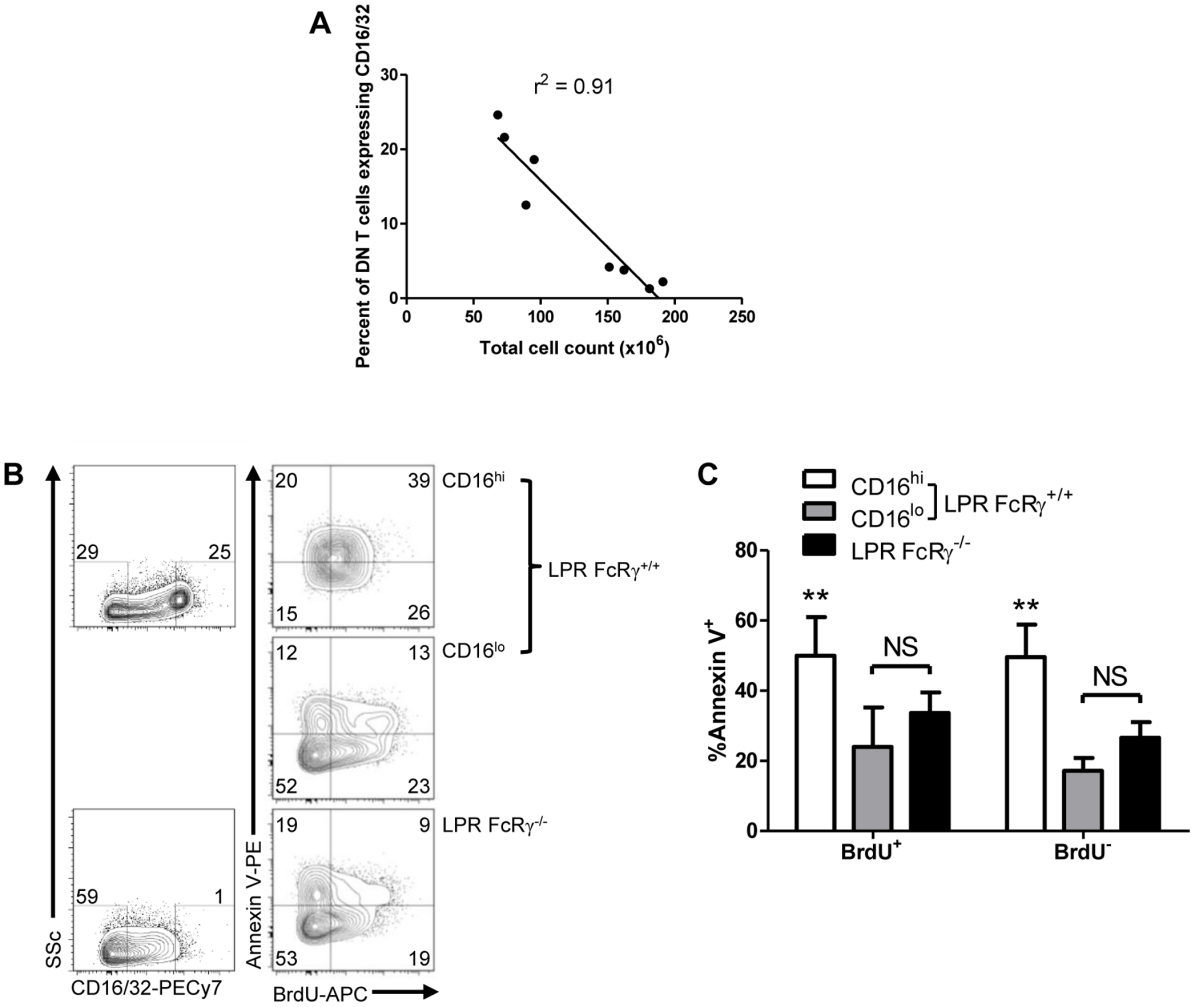
Fc receptor γ-expressing DN T cells are lost with disease progression in LPR mice and have an increased rate of apoptosis *ex vivo*. **A.** Splenocytes of young (≤12 weeks, n = 4) and older (≥16 weeks, n = 4) female LPR mice were counted and stained for expression of CD4, CD8, NK1.1, TCRβ, and CD16 and then examined by flow cytometry. The percentage of DN T cells expressing CD16 was examined as a function of total spleen cell count; linear regression r^2^ = 0.91, p = 0.0002. **B.** LPR FcRγ^+/+^ (n = 5) and LPR FcRγ^−/−^ mice (n = 5) aged 8 weeks were fed BrdU for 6 days. Their splenocytes were then stained for expression of CD4, CD8, NK1.1, TCRβ, annexin V and CD16, fixed and stained for BrdU incorporation. They were then examined by flow cytometry. Left panels show expression of CD16 versus side light scatter in LPR FcRγ^+/+^ (top) and LPR FcRγ^−/−^ DN T cells (bottom); CD16^hi^ and CD16^lo^ gates are indicated, and the numbers above each gate indicate the percentage of DN T cells falling into the indicated gates. Right panels show representative BrdU and annexin V staining in LPR FcRγ^−/−^ DN T cells (bottom) and in the CD16^lo^ and CD16^hi^ subsets of LPR FcRγ^+/+^ DN T cells (middle and top panels, respectively). Numbers inside contour plots reflect the percentage of gated cells falling into each quadrant. **C.** The percentages of DN T cells staining with annexin V in LPR FcRγ^−/−^ mice and in the CD16^hi^ and CD16^lo^ subsets of LPR FcRγ^+/+^ mice are presented with respect to BrdU incorporation. Two-way ANOVA p<0.0001; **Bonferroni post tests p<0.01 compared with either CD16^lo^ or LPR FcRγ^−/−^ DN T cells amongst both BrdU^+^ and BrdU^−^ DN T cells.

In order to understand the relationship between FcRγ expression in DN T cells and the development of lymphoproliferative disease, we compared DN T cell FcRγ/CD16 expression in younger (∼12 weeks of age) and older (∼16 weeks of age) LPR mice and observed a loss of this subset as lymphocytes accumulated (**Fig. 3A**, r^2^ = 0.91). This finding suggested that FcRγ-expressing DN T cells might be proliferating more slowly and/or dying more rapidly than DN T cells not expressing this molecule in LPR mice.

To investigate the contributions of proliferation and cell death further, 8 week old LPR FcRγ^+/+^ and LPR FcRγ^−/−^ mice were fed BrdU in the drinking water for 6 days. Their splenocytes were then stained for TCRβ, CD4, CD8, NK1.1, CD16 expression, annexin V binding, and BrdU incorporation and analyzed by flow cytometry. As shown in **Fig. 3B**, 65% of CD16^hi^ DN T cells in LPR FcRγ^+/+^ mice incorporated BrdU whereas only 36% of LPR FcRγ^+/+^ CD16^lo^ and 28% of LPR FcRγ^−/−^ DN T cells did so, indicating a higher rate of proliferation in CD16^hi^ DN T cells *in vivo*. Interestingly, a significantly increased rate of apoptosis was also seen in both BrdU^−^ and BrdU^+^ CD16^hi^ LPR FcRγ^+/+^ DN T cells compared with either CD16^lo^ DN T cells from the same animal or LPR FcRγ^−/−^ DN T cells. (**Fig. 3C**, two-way ANOVA p<0.0001). Althoughthe proportion of live, proliferating (annexin V^−^ BrdU^+^) cells was similar amongst CD16^hi^ and CD16^lo^ LPR FcRγ^+/+^ and LPR FcRγ^−/−^ DN T cells (**Fig. S1A** and **Fig. S1B**, p = NS), a much larger proportion of both proliferated and unproliferated CD16^hi^ DN T cells had become apoptotic in comparison with both CD16^lo^ and LPR FcRγ^−/−^ DN T cells (**Fig. S1A** and **Fig. 3B–C**). These observations suggest that the loss of CD16^hi^ DN T cells with increasing lymphocyte accumulation is likely due to a higher rate of apoptosis within this population.

### The Ability of LPR DN T Cells to Control Lymphoproliferative Disease Depends on Both FcRγ and Fas

**Figure 4:**
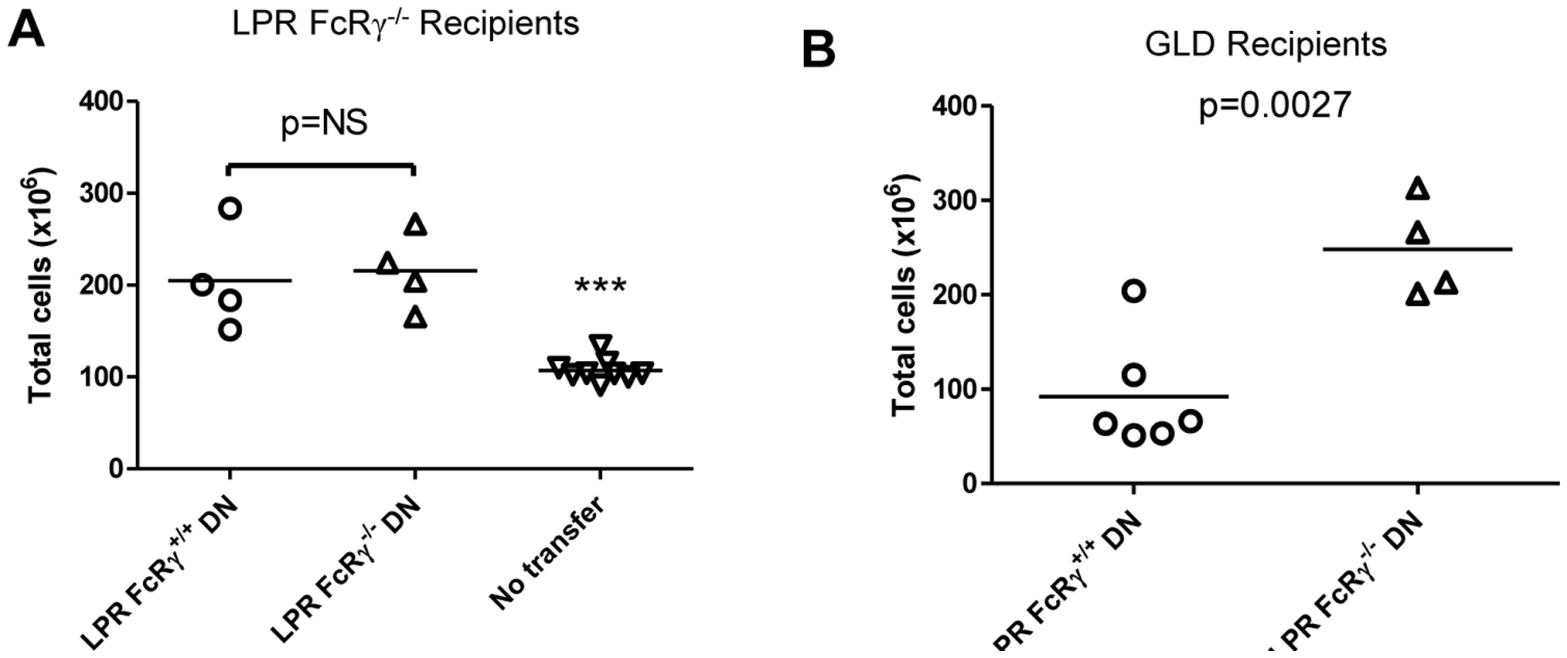
Control of lymphoproliferative disease by LPR DN T cells requires both Fas and FcRγ. **A.** B6.LPR.FcRγ^−/−^ mice aged 4 weeks received two i.v. injections, two weeks apart, each of either B6.LPR.FcRγ^+/+^ DN T cells (n = 4) or B6.LPR.FcRγ^−/−^ DN T cells (n = 4). Cells derived from one-two donor mice (generally 10–20×10^6^ cells per dose) were used for each injection, ensuring that an equivalent number of B6.LPR.FcRγ^−/−^ and B6.LPR.FcRγ^+/+^ DN T cells were transferred on each occasion. After a further 4 weeks, total spleen and lymph node cell counts were determined. Total cell counts for a group of 8 week old uninjected B6.LPR.FcRγ^−/−^ mice (n = 9) are shown for comparison. One-way ANOVA p<0.0001; ***Bonferroni post tests p<0.001 for the comparison of untreated mice to both groups of treated mice; Bonferroni post test p = NS for the comparison of the two groups of treated animals. **B.** B6.GLD mice aged 6 weeks were given either B6.LPR.FcRγ^+/+^ or B6.LPR.FcRγ^−/−^ DN T cells. Cells derived from one-two donor mice were used for each injection (between 10–20×10^6^ cells), ensuring that an equivalent number of B6.LPR.FcRγ^−/−^ and B6.LPR.FcRγ^+/+^ DN T cells were transferred. After 3 weeks, total splenocyte counts were determined. Unpaired t test, p = 0.0027.

Since FcRγ-expressing 2C TCR transgenic DN T cells possess regulatory function [25], and LPR FcRγ^−/−^ mice exhibit exacerbated lymphocyte accumulation in comparison with LPR FcRγ^+/+^ mice [26], we hypothesized that FcRγ expression in the DN T cell compartment of LPR mice might confer similar properties on these cells. We therefore chose to assess whether FcRγ-expressing LPR DN T cells might have a regulatory effect when transferred to LPR FcRγ^−/−^ mice. Hence, DN T cells were purified from LPR FcRγ^+/+^ or LPR FcRγ^−/−^ mice and transferred i.v. to LPR FcRγ^−/−^ recipients at 4 weeks of age, prior to the onset of lymphoproliferation. A second similar treatment was given 2 weeks later. The total DN T cell yield from 1–2 donor mice aged 8–12 weeks was transferred to each LPR FcRγ^−/−^ recipient on each occasion, ensuring that mice received an equivalent number (∼10–20×10^6^ DN T cells per dose) of either LPR FcRγ^+/+^ or LPR FcRγ^−/−^ DN T cells. After a further 4 weeks, spleen and lymph node cell counts were obtained, but although recipients of both types of DN T had increased total cell counts in comparison with LPR FcRγ^−/−^ mice that did not receive any cells (one way ANOVA p<0.0001; Bonferroni post tests p<0.001), no differences were observed between mice receiving LPR FcRγ^+/+^ and LPR FcRγ^−/−^ DN T cells (**Fig. 4A**).

Previous studies have demonstrated that Fas-FasL interaction is critical for DN T cells to exert their regulatory effects [4], [20], [28], [29]. We hypothesized that an FcRγ-dependent regulatory function of LPR DN T cells might also require Fas-FasL interaction. To determine whether the inability to inhibit lymphoproliferation might be due to the lack of functional Fas expression in LPR mice, we tested the role of FcRγ in LPR DN T cell function in GLD mice. These animals develop a lymphoproliferative disease identical to that of LPR mice as a result of the absence of functional FasL. Six-week-old GLD mice were treated with either LPR FcRγ^+/+^ or LPR FcRγ^−/−^ DN T cells. Each mouse received a single dose of DN T cells from 1–2 donor mice (∼10–20×10^6^ cells), again ensuring an equivalent number of either LPR FcRγ^−/−^ or LPR FcRγ^+/+^ DN T cells were transferred. In this instance, we observed a reduction in total lymphocyte counts in recipients of LPR FcRγ^+/+^ DN T cells compared with recipients of LPR FcRγ^−/−^ DN T cells (**Fig. 4B**, p = 0.0027). Thus, LPR DN T cells can inhibit lymphocyte accumulation in an FcRγ-dependent manner provided Fas is available on the expanding lymphocytes.

### LPR DN T Cells Suppress Allogeneic Immune Responses in an FcRγ- and Fas-dependent Manner

**Figure 5:**
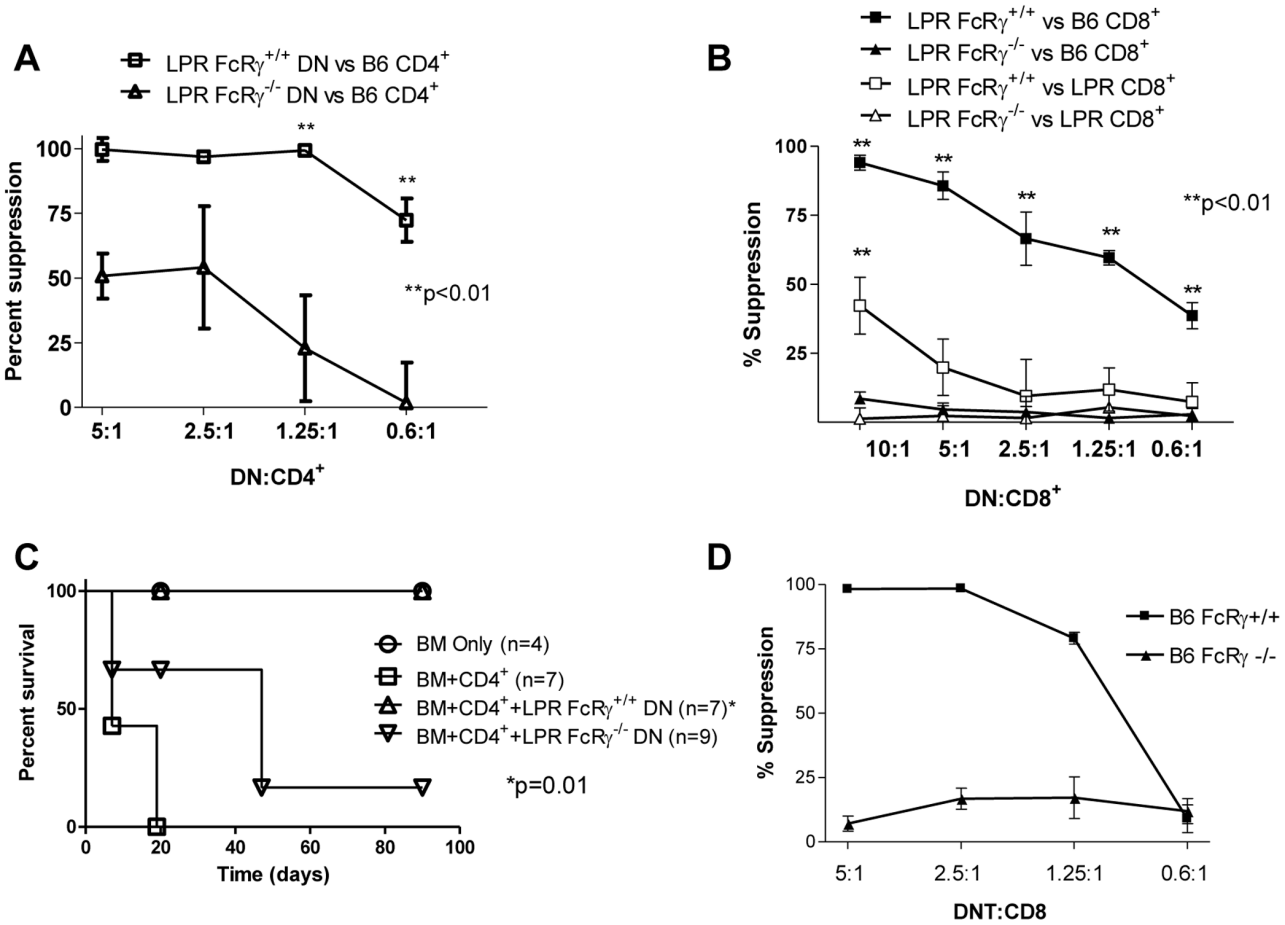
FcRγ expression by LPR DN T cells is required for their regulatory function toward Fas-expressing syngeneic T cells. **A.** B6 CD4^+^ T cells (10^4^/well) were co-cultured with irradiated CB6F1 splenocytes (10^5^/well) and IL-2. Purified B6.LPR.FcRγ^+/+^ or B6.LPR.FcRγ^−/−^ DN T cells were added in varying ratios. After 4 days, ^3^H-thymidine (1 µCi/well) was added. After 18 h, ^3^H-thymidine uptake was determined, and percent suppression was calculated. Two-way ANOVA p<0.0001; Bonferroni post-tests **p<0.01. Data are from one of three experiments with similar results. **B.** B6 or B6.LPR.FcRγ^+/+^ CD8^+^ T cells (10^4^/well) were cultured as in C. Percent suppression was calculated. Two-way ANOVA p<0.0001 for the effect of FcRγ on suppression of B6 CD8^+^ T cells; p = 0.0043 for the effect of FcRγ on suppression of B6.LPR CD8^+^ T cells. Bonferroni post-tests **p<0.01. Data are from one of three experiments with similar results. **C.** Male CB6F1 mice were lethally irradiated and reconstituted with 2×10^6^ TCD BM alone (BM Only) or with 10^6^ B6 CD4^+^ T cells (BM+CD4^+^), with or without 5×10^6^ B6.LPR.FcRγ^+/+^ or B6.LPR.FcRγ^−/−^ DN T cells. Mice losing >25% of their body weight were sacrificed. *Log rank test p = 0.01 for the comparison of mice treated with B6.LPR.FcRγ^+/+^ and B6.LPR.FcRγ^−/−^ DN T cells. Data are derived from two independent experiments, each with 2–5 mice per group. **D.** Naive B6 CD8^+^ T cells were used as responders and stimulated by irradiated bm1 splenocytes. Varying numbers of DN T cells isolated from spleens of bm1 splenocyte-treated B6.FcRγ^+/+^ (squares) or B6.FcRγ^−/−^ (triangles) mice were added to the MLR cultures as putative suppressor cells. Cell proliferation was measured by ^3^H-thymidine incorporation. The data are expressed as percent inhibition of proliferation as compared with the controls to which no putative suppressor cells were added. Data points are the mean +/− SD of triplicate wells and are derived from one of three independent experiments.

To determine further the importance of FcRγ expression in the regulatory function of LPR DN T cells in an allogeneic setting, B6 CD4^+^ T cells were co-cultured with allogeneic CB6F1 splenocytes in the presence of either LPR FcRγ^+/+^ or LPR FcRγ^−/−^ DN T cells in varying ratios. After 3 days, proliferation of CD4^+^ T cells was determined and the percent suppression calculated. As shown in **Fig. 5A**, LPR FcRγ^−/−^ DN T cells had a reduced ability to suppress the alloantigen-driven proliferation of B6 CD4^+^ T cells in comparison with LPR FcRγ^+/+^ DN T cells. We observed a similar phenomenon when B6 CD8^+^ T cells were used as responders (**Fig. 5B** **, closed symbols**; two way ANOVA p<0.0001 for the effect of FcRγ; Bonferroni post test p<0.01 at all ratios). Thus, LPR DN T cells can inhibit syngeneic Fas^+^ T cells responding to alloantigens in an FcRγ-dependent manner.

To validate the requirement of Fas expression on responder cells, the same experiment was performed using purified CD8^+^ T cells from Fas mutant LPR mice. Consistent with the finding in autologous model (**Fig. 4**), LPR FcRγ^+/+^ DN T cells exhibited only very limited inhibition of Fas-deficient responder cells (**Fig. 5B** **, open squares**) compared with Fas-sufficient responder cells (**Fig. 5B** **, closed squares**, two-way ANOVA p<0.0001 for the effect of responder cell Fas expression; Bonferroni post tests p<0.01 at all but the 0.6∶1 ratio). Deficiency of FcRγ also impaired the ability of LPR DN T cells to suppress Fas-deficient responder cells, but only at a high DN:CD8^+^ T cell ratio (**Fig. 5B** **, open squares and triangles**, two-way ANOVA p = 0.004, Bonferroni post test p<0.01 at the 10∶1 ratio, p = NS at other ratios). These data therefore indicate that alloantigen-reactive T cells that lack Fas cannot effectively be suppressed by LPR DN T cells. As was seen when LPR FcRγ^+/+^ or LPR FcRγ^−/−^ DN T cells were administered to GLD mice, FcRγ expression by LPR DN T cells is required for their ability to suppress Fas-sufficient alloantigen-reactive T cells effectively.

To test the importance of FcRγ in the regulatory function of LPR DN T cells during an alloimmune response *in vivo*, CB6F1 mice were lethally irradiated and reconstituted with 2×10^6^ TCD B6 BM cells alone (BM only, n = 4) or with 1×10^6^ B6 CD4^+^ T cells, without (BM+CD4^+^, n = 7) or with 5×10^6^ LPR FcRγ^+/+^ or LPR FcRγ^−/−^ DN T cells (BM+CD4^+^+LPR FcRγ^+/+^ or LPR FcRγ^−/−^ DN, n = 7 and n = 9, respectively). They were then followed daily for survival. As shown in **Fig. 5C**, LPR FcRγ^+/+^, but not LPR FcRγ^−/−^, DN T cells could rescue recipients from lethal GVHD (log rank p = 0.01). Taken together, these data illustrate that expression of FcRγ in LPR DN T cells is critical for their ability to regulate alloantigen-reactive Fas^+^ T cells.

Since FcRγ expression in LPR DN T cells is correlated with surface expression of CD16 [26], we attempted to sort DN T cells into CD16^hi^ and CD16^lo^ populations for use in suppression assays and *in vivo* experiments. These attempts were not successful, possibly due to the high rate of apoptosis in the CD16^hi^ compartment (**Fig. 3B–C** and [26]). Therefore, we sought confirmation of our observations on the role of FcRγ in a different population of DN T cells. To this end, we infused Fas-sufficient FcRγ^+/+^ and FcRγ^−/−^ B6 mice with allogeneic bm1 splenocytes and, 7 days later, enriched DN T cells from their secondary lymphoid organs and used them as putative suppressor cells. Naïve FcRγ^+/+^ B6 CD8^+^ T cells were stimulated by irradiated bm1 splenocytes in the presence of absence of varying numbers of FcRγ^+/+^ or FcRγ^−/−^ DN T cells. As shown in **Fig. 5D**, B6 FcRγ^−/−^ DN T cells were unable to suppress the proliferation of B6 CD8^+^ T cells responding to alloantigens, unlike their FcRγ-sufficient counterparts. Thus, as with LPR DN T cells and 2C TCR transgenic DN T cells [25], B6 DN T cells also require expression of FcRγ in order to act as regulatory T cells.

### Suppression of Fas-sufficient T cells by LPR DN T Cells Results in the Fas-mediated Cytolysis of Responding T Cells

**Figure 6:**
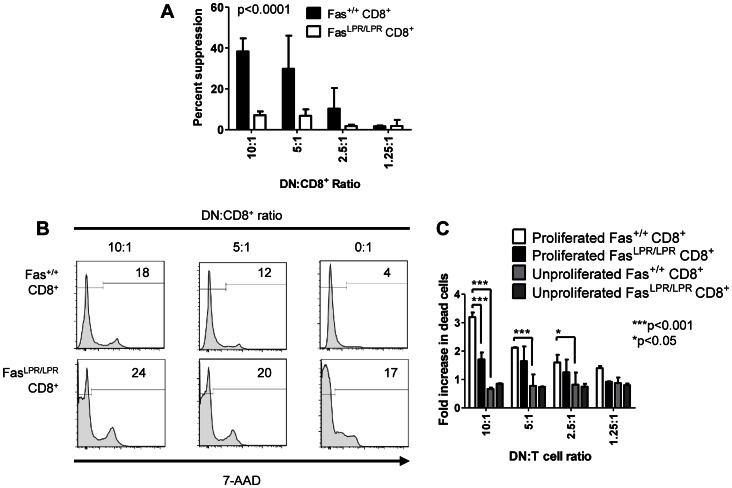
CD8^+^ T cells proliferating in response to alloantigen are selectively killed by LPR DN T cells via the Fas pathway. CD8^+^ T cells (10^5^/well) from B6 (Fas^+/+^) or B6.LPR (Fas^LPR/LPR^) were labelled with CFSE and cocultured with irradiated CB6F1 splenocytes and IL-2 for 5 days without or with LPR DN T cells in the indicated ratios. After 5 days, the cultures were stained with anti-CD8-APC and 7-AAD and analyzed by flow cytometry. **A.** The percentage of undivided cells (CFSE^hi^) was used to determine the percent suppression at each DN:CD8^+^ T cell ratio. Two-way ANOVA p<0.0001 for the effect of CD8^+^ T cell Fas expression. **B.** Representative histograms of 7-AAD staining gated on proliferated (CFSE-diluted) Fas^+/+^ (top row) and Fas^LPR/LPR^ (bottom row) CD8^+^ T cells at the indicated DN:CD8^+^ ratios. Numbers inside histograms are the percentages of cells falling in the 7-AAD^+^ gate. **C.** The fold increase in cell death for proliferated Fas^+/+^ (white bars), proliferated Fas^LPR/LPR^ (black bars), unproliferated Fas^+/+^ (light grey bars), proliferated Fas^LPR/LPR^ (dark grey bars) CD8^+^ T cells is shown. Two-way ANOVA p<0.0001; Bonferrroni post-tests ***p<0.001 and *p<0.05. Data are derived from two independent experiments each with duplicate wells.

Much of the literature on DN Tregs demonstrates that these cells inhibit T cell responses via the Fas pathway (reviewed in [30], [31]), and this is true for LPR DN T cells as well [4], [29]. While Fas-mediated cytolysis of activated T cells is well described, there are also data to show that Fas ligation on naïve T cells can inhibit their initial activation without causing apoptosis [32], [33]. We recently demonstrated that proliferating, alloreactive Fas^+^ CD4^+^ T cells were selectively killed by LPR DN T cells during a CFSE suppression assay [29]. To determine whether this is also true for CD8^+^ T cells, we co-cultured CFSE-labelled B6 or LPR CD8^+^ T cells with irradiated CB6F1 splenocytes and IL-2. LPR DN T cells were added to the cultures in varying ratios and after 5 days, CFSE dilution and 7-AAD staining were jointly examined in CD8^+^ T cells by flow cytometry. As shown in **Fig. 6A**, the proliferation of Fas-expressing, but not Fas-deficient, CD8^+^ T cells was suppressed by LPR DN T cells (two-way ANOVA p<0.0001).

We then examined CD8^+^ T cell death amongst unproliferated (CFSE^hi^) and proliferated (CFSE-diluted) cells. As shown in **Fig. 6B–C**, there was a dose-dependent increase in 7-AAD staining amongst proliferated Fas^+^ but not proliferated LPR CD8^+^ T cells. In contrast, LPR DN T cells did not cause an increase in cell death amongst unproliferated B6 or LPR CD8^+^ T cells (**data not shown** and **Fig. 6C**, two-way ANOVA p<0.0001). Hence these data demonstrate that during suppression of Fas-sufficient CD8^+^ T cells, LPR DN T cells selectively cause the death of proliferating cells, while they are unable to suppress or kill LPR T cells.

## Discussion

In this study, we showed that FcRγ expression in LPR DN T cells is critical for their ability to suppress CD4^+^ and CD8^+^ T cells responding to auto- and allo-antigens, and that this suppression requires FasL-Fas interactions. FcRγ-deficient LPR DN T cells showed greatly reduced regulatory function toward B6 CD4^+^ and CD8^+^ T cells responding to alloantigens *in vitro* and attenuate CD4^+^ T cell induced GVHD (**Fig. 5**). In our previous study [25], L^d^-specific FcRγ^+/+^ DN T cells, but not FcRγ^−/−^ DN T cells, could block the rejection of L^d+^ allografts by L^d^-specific CD8^+^ T cells in single class I MHC-mismatched recipients. Thus, the data presented here confirm and extend the previous report and indicate that FcRγ expression is critical for LPR DN T cells to suppress immune responses toward alloantigens *in vitro* and *in vivo*. Although we did not formally examine an association of FcRγ with the TCR in LPR DN T cells, other T cells expressing FcRγ incorporate it into the TCR complex in lieu of CD3ζ [13], [14], [25], [34], [35]. Our data therefore suggest that TCR-derived signals are likely to be important for the function of LPR DN T cells as Tregs.

In addition to suppressing alloreactive T cells, we found that adoptively transferred FcRγ-sufficient LPR DN T cells inhibited the progressive accumulation of lymphocytes in GLD mice, whereas FcRγ^−/−^ LPR DN T cells failed to do so (**Fig. 4B**). This finding suggests that FcRγ expression in LPR DN T cells can also play a part in controlling autoimmune lymphoproliferative disease. In contrast to the effect on GLD mice, adoptive transfer of the same number of FcRγ-sufficient LPR DN T cells failed to inhibit the accumulation of T cells in FcRγ^−/−^ LPR mice (**Fig. 4B**). In fact, transfer of LPR DN T cells, regardless of their FcRγ genotype, resulted in an increased total lymphocyte count in LPR.FcRγ^−/−^ mice (**Fig. 4B** **, right column**). This observation is consistent with the recent finding that DN T cells can drive T cell accumulation in this Fas-deficient context [6]. Since GLD mice express functional Fas whereas LPR mice do not, our observations further indicate that FcRγ-mediated signaling is required for LPR DN T cells to provide a FasL-mediated death signal toward target cells. Consistent with this notion, we found that LPR FcRγ^+/+^ DN T cells could inhibit B6 but not LPR CD8^+^ T cell proliferation in response to alloantigen stimulation (**Fig. 6A**). Furthermore, suppression of CD8^+^ T cells was mainly due to the killing of proliferated CD8^+^ T cells (**Fig. 6B–C**). LPR DN T cells are known to express FasL constitutively [36], [37]. However, those studies did not examine differences in FasL surface expression in response to TCR stimulation, which has been shown to mediate FasL translocation from intracellular compartments to the cell surface [38], [39]. We have recently demonstrated that this is also true for LPR DN T cells, in that the combination of TCR stimulation and autocrine IFNγ secretion and signaling was required for efficient externalization of presynthesized FasL on the cell surface, leading to regulatory function *in vitro* and *in vivo* [29]. The present study extends these findings by illustrating that the FasL-mediated regulatory function of LPR DN T cells also requires FcRγ. We have not tested how FcRγ might participate in this process, but it is possible that a signal arising from FcRγ, but not CD3ζ, might be required to trigger sufficient IFNγ release to cause FasL externalization; alternatively, FcRγ-derived signals might be required independently of IFNγ secretion and signaling. These hypotheses will require further experimental testing.

In addition to Fas expression, another major difference between GLD and LPR FcRγ^−/−^ mice is that the former express FcRγ. Since FcRγ is expressed by NK cells, myeloid cells, DCs, and macrophages, we also treated LPR FcRγ^−/−^ mice with CD3^−^CD19^−^ cells or activated NK cells from either LPR FcRγ^+/+^ or LPR FcRγ^−/−^ mice, but we did not observe any reduction in lymphocyte accumulation in LPR FcRγ^−/−^ mice treated with LPR FcRγ^+/+^ NK cells or CD3^−^CD19^−^ cells (**Fig. S2A–B**). Similarly, although CD4^+^Foxp3^+^ Tregs are known to control lymphoproliferation and autoimmunity [40], examination of LPR lymph node CD4^+^ T cells revealed that FcRγ-expressing cells were distinct from the Foxp3^+^ population (**Fig. S2C**). Thus, it appears that the inability of FcRγ^+/+^ LPR DN T cells to inhibit the progression of lymphocyte accumulation in FcRγ^−/−^ LPR mice is due to the lack of functional Fas rather than a functional impairment of other FcRγ-expressing cells in recipient mice.

In addition to requiring expression of FcRγ in order to suppress the proliferation of Fas^+^ CD4^+^ and CD8^+^ T cells (a cell-extrinsic function of FcRγ), LPR DN T cells also showed cell-intrinsic differences in the propensity to accumulate *in vitro* and *in vivo* on the basis of their FcRγ genotype. Proliferation of FcRγ^+^ DN T cells was significantly reduced compared with FcRγ^−^ DN T cells *in vitro*, and the former accumulated to a much lesser extent after adoptive transfer into B6 SCID mice (**Fig. 2**). This finding is consistent with our recent observation that FcRγ-expressing DN T cells show a high rate of apoptosis *in vivo* compared with FcRγ deficient DN T cells (**Fig. 3** and [26]). To our knowledge, FcRγ signaling in T cells has not previously been associated with apoptosis, although it has been described to mediate the death of NK cells and granulocytes in certain circumstances [41]–[43]. Further, while TCRs that contain FcRγ have been shown to result in hyperresponsiveness to TCR signaling [19], only limited data exist on specific differences in T cell function resulting from FcRγ incorporation in the TCR complex [18], [25].

Given the differing roles played by FcRγ in this study, it is interesting to note that a population of short-lived Foxp3^+^ Tregs induced by donor-specific splenocyte infusion and capable of inhibiting allograft rejection has recently been identified [44]. These Tregs expressed T cell immunoglobulin and mucin domain-3 (TIM-3), which is commonly expressed on activated Th1 and Th17 cells [45]–[47]. Interestingly, although TIM-3^+^ Tregs could more potently inhibit effector T cells than TIM-3^−^ Tregs *in vitro* and preferentially infiltrated skin allografts, they expressed PD-1 and were susceptible to galectin-9-mediated death and actually exhibited reduced potency upon adoptive transfer [44]. These data suggest that potent Tregs are carefully controlled by mechanisms that promote both their regulatory function and their clearance *in vivo*. Our data suggest that FcRγ might play a similar role in DN T cells, although further work will be required to investigate this function of FcRγ.

DN T cells in LPR mice are a heterogeneous population. A recent study has suggested that LPR DN T cells can stimulate, rather than inhibit the expansion of CD4^+^ and CD8^+^ T cells within LPR mice by promoting the expansion of the follicular dendritic cell network [6]. Currently, no marker that can distinguish regulatory and non-regulatory DN T cells has yet been identified. Together with our previous study [25], the data presented here suggest that FcRγ expression by DN T cells is required for these cells to exert regulatory function. Our previous work also showed that FcRγ was the most differentially expressed gene between regulatory DN T cell clones and their non-regulatory mutants [24]. Collectively, these findings suggest that FcRγ might serve as a novel marker for DN Tregs. It will be of interest to determine whether human DN T cells [48], [49] express FcRγ, and whether it confers regulatory function upon them as well. If so, it will be important to examine whether FcRγ-expressing DN T cells are a unique lineage developing under different stimuli, or whether they can differentiate from FcRγ^−^ DN T cells. These studies could lead to the identification of DN Treg-specific lineage specification factors, and therefore represent a critical direction for future research.

In conclusion, FcRγ exhibited two distinct functions in LPR DN T cells: cell-extrinsic regulation of Fas^+^ T cell proliferative responses to allo- and auto-antigen, and cell-intrinsic regulation of DN T cell accumulation within the LPR setting. Additional studies will be required to better define the role of FcRγ in DN T cell regulatory function and whether FcRγ is a universal marker of regulatory DN T cells.

## Materials and Methods

### Ethics Statement

Animals were housed in the Toronto Medical Discovery Tower under specific pathogen-free conditions. The animal use protocols (#322 and #741) were approved by the University Health Network Animal Care Committee. Animal care was conducted in accordance with the policies and guidelines of the Canadian Council on Animal Care and the Province of Ontario’s Animals for Research Act.

### Mice

C57BL/6 (B6, H-2^b^),BALB/c (H-2^d^), and B6.H-2K^bm1^ (bm1, #000368) were obtained from Jackson Laboratories. B6 mice with the LPR mutation in Fas (#000482), with the GLD mutation in FasL (#001021), and severe combined immunodeficiency B6 mice (B6.SCID, #001913) were purchased from Jackson Laboratories (Bar Harbor, ME) and bred in-house. FcRγ gene-targeted B6 mice (#000583) were obtained from Taconic (Hudson, NY). LPR FcRγ^−/−^ mice were generated by breeding the appropriate strains to homozygosity. Mice were housed in specific pathogen-free conditions in the Toronto Medical Discovery Tower animal facility. The Animal Use Protocol (#741) was approved by the institutional Animal Care Committee.

### Antibodies and Flow Cytometry

The following mAbs were obtained from BioLegend (San Diego CA): FITC-conjugated anti-CD3, anti-CD4, anti-CD8, anti-TCRβ, and anti-NK1.1; PE-conjugated anti-CD3, anti-CD4, anti-CD8, and anti-NK1.1; PerCP-Cy5.5-conjugated anti-NK1.1; PE-Cy7-conjugated anti-CD4; allophycocyanin-conjugated anti-Foxp3, anti-CD4 and anti-CD8; allophycocyanin-Cy7-conjugated anti-CD4 and anti-CD8; PE-Cy5-conjugated anti-CD8; Alexa Fluor 700-conjugated anti-TCRβ; and purified anti-CD28. Purified rabbit anti-mouse FcRγ mAb was obtained from Upstate Cell Signaling Solutions (Lake Placid NY). PE-conjugated donkey anti-rabbit F(ab′)_2_ was obtained from eBioscience. Purified anti-CD3 mAb was prepared from the 145-2C11 hybridoma (ATCC, Manassas VA) in house. PE-conjugated annexin V was from BioLegend.

Fixation and intracellular staining was performed using either the eBioscience intracellular staining kit. Flow cytometry was performed on an LSR II (BD Biosciences), an Accuri C6 (BD Biosciences), or an EPICS-XL (Beckman-Coulter).

### Cell Purification

T cell depleted bone marrow (TCD BM) (>99% T cell free) was obtained by treating erythrocyte-free BM cells with anti-Thy1.2 ascites (TIB-107 hybridoma, ATCC, Manassas, VA) and Low-Tox M rabbit complement (Cedarlane Labs, Burlington ON). CD4^+^ T cells (>90% pure) were purified from B6.Thy1.1 spleen and lymph node cells with anti-CD4 microbeads (Miltenyi Biotec, Auburn CA). To obtain DN T cells for bone marrow transplantation (BMT), LPR FcRγ^+/+^ or LPR FcRγ^−/−^ mice aged 8–12 weeks were infused with 40×10^6^ CB6F1 splenocytes to activate and expand DN T cells [4], [20]. After 7 days, DN T cells were purified by removing CD4^+^, CD8^+^, NK1.1^+^, CD19^+^, CD11b^+^, CD11c^+^, γδTCR^+^, and Ter119^+^ populations with PE-conjugated mAbs and anti-PE microbeads (Miltenyi Biotec; resulting population >99% PE^−^ and 75–90% TCRβ^+^CD4^−^CD8^−^NK1.1^−^ cells). To obtain DN T cells from B6 mice, T cells were enriched over nylon wool columns followed by depletion of CD4^+^ and CD8^+^ populations using anti-CD8 (clone 3.168.8) and anti-CD4 (clone RL172) IgM antibodies followed by Low-Tox M rabbit complement (Cedarlane). Further enrichment of DN T cells was perfomed if required using biotinylated anti-CD3 (eBioscience) and anti-biotin microbeads (Miltenyi). Cell number was adjusted based DN T cell purity to ensure that a consistent number of DN T cells were used.

NK cells were purified by depleting CD3^+^ cells from LPR FcRγ^+/+^ and LPR FcRγ^−/−^ splenocytes with anti-CD3 microbeads (Miltenyi Biotec) and then enriching DX5^+^ cells with anti-DX5 microbeads. The purified population was >90% NK1.1^+^. Activated NK cells were obtained by culturing LPR FcRγ^+/+^ or LPR FcRγ^−/−^ splenocytes in 50 mL cultures containing 100 U/mL IL-2. The media was changed on day 3 or 4. On day 7, NK cells were purified from the cultures using the method just described.

DN T cells and CD3^−^CD19^−^ cells were jointly purified from LPR FcRγ^+/+^ and LPR FcRγ^−/−^ spleen and lymph node cell suspensions by first depleting CD4^+^, CD8^+^, and CD19^+^ cells with PE-conjugated antibodies and anti-PE microbeads. Anti-CD3 microbeads were then used to separate the negative fraction into DN T cells and CD3^−^CD19^−^ components. The CD3^+^ fraction contained >90% DN T cells (the remainder included some NK T cells). The CD3^−^ fraction was >90% free of CD3^+^ cells. Additional columns were used as required to achieve these levels of purity.

### BMT and GVHD Induction

Male CB6F1 mice aged 6–10 weeks received two 6.5 Gy doses of γ-irradiation (>4 h apart) in a Gammacell 40 ^137^Cs irradiator (MDS Nordion, Ottawa ON) and were infused with 2×10^6^ TCD BM alone or with 10^6^ CD4^+^ T cells, with/without 5×10^6^ purified DN T cells, as we previously reported [29]. Survival was monitored daily. Weights and clinical scores [50] were determined 2–3 times weekly. Moribund mice (score >6 or weight loss >25%) were sacrificed.

### CFSE Suppression Assay

CD4^+^ or CD8^+^ T cells (10^7^ cells/mL) were incubated (10 minutes, 37°C) in PBS containing 1 µM CFSE (Invitrogen, Carlsbad CA), followed by quenching with FBS (Gibco, Carlsbad CA). Cells were washed in α-minimum essential medium with 10% FBS, 50 mM β-mercaptoethanol, 0.1 mg/mL penicillin and 0.1 mg/mL streptomycin (CM). 10^5^ CFSE labelled cells were cultured for 5 days with 2×10^5^ irradiated (20 Gy) CB6F1 splenocytes and 50 U/mL recombinant human IL-2 (Proleukin, Chiron Corporation, Emeryville CA). Purified DN T cells (not CFSE labelled) were added in varying ratios. Cells were stained with 7-AAD (Sigma-Aldrich) prior to analysis to permit identification of dead cells. Percent suppression was calculated using the formula: [(%CFSE^hi^ (DN+CD4^+^) - %CFSE^hi^ (CD4^+^ only))/(100-%CFSE^hi^ (CD4^+^ only))] x 100%. The live:dead ratio was determined by dividing the percentage of 7-AAD^−^ cells by the percentage of 7-AAD^+^ cells in the CFSE^hi^ and CFSE-diluted gates.

### ^3^H-thymidine Suppression and Proliferation Assays

Purified CD4^+^ or CD8^+^ B6 or LPR FcRγ^+/+^ T cells were cultured (10^4^/well) with irradiated CB6F1 splenocytes (10^5^/well) and 50 U/mL IL-2 in 200 µL cultures. Purified LPR FcRγ^+/+^ or LPR FcRγ^−/−^ DN T cells were added in varying ratios as putative suppressor cells. Cultures containing the same numbers of DN T cells and CB6F1 splenocytes, but without responder T cells, were run in parallel. After 4 days, 1 µCi/well ^3^H-thymidine (Perkin-Elmer, Woodbridge ON) was added to all cultures and after a further 18 h, the cultures were harvested and the cpm of retained DNA, determined on a TopCount NXT (Perkin-Elmer), was used to calculate percent suppression according to the following formula: [(cpm (T alone) – [cpm (T+DN) – cpm(DN alone)]/(cpm (T alone)] x 100%.

Proliferation of DN T cells *in vitro* was assessed by culturing *in vivo*-preactivated (by infusion of 4×10^7^ CB6F1 splenocytes 7 days prior) DN T cells for 3 days with irradiated CB6F1 splenocytes and 50 U/mL IL-2, followed by the addition of 1 µCi/well ^3^H-thymidine. After 18 h, cultures were harvested and the cpm of retained DNA, determined on a TopCount NXT (Perkin-Elmer), was reported as an index of proliferation.

### Adoptive Transfer Studies and in vivo Cell Tracking

LPR FcRγ^+/+^ and LPR FcRγ^−/−^ recipient mice for adoptive transfer studies were 4 weeks of age. CB6F1 recipients of BMT were 6–10 weeks of age. B6.GLD recipient mice were 6 weeks of age. Donor LPR FcRγ^−/−^ and LPR FcRγ^+/+^ mice were generally 8–12 weeks of age to ensure the onset of DN T cell accumulation prior to use.

Proliferation of LPR FcRγ^+/+^ CD8^+^ T cells in B6.SCID mice was assessed by labelling purified CD8^+^ T cells with CFSE at a concentration of 5 µM as described above. The cells were then injected (2.5×10^6^/mouse) via the lateral tail vein alone or with 10^7^ purified LPR FcRγ^−/−^ or LPR FcRγ^+/+^ DN T cells. After 24 h, splenocytes were stained with anti-CD3 and anti-CD8 mAbs and examined for CFSE dilution in the CD3^+^CD8^+^ gate by flow cytometry.

### Measurement of Cell Cycling and Apoptosis with BrdU and Annexin V Labeling

LPR FcRγ^+/+^ and LPR FcRγ^−/−^ mice aged 8 weeks were fed BrdU in the drinking water at 0.8 mg/mL for 6 days. Their erythrocyte-free splenocytes were then stained for expression of TCRβ, CD4, CD8, NK1.1. The cells were washed and then stained with PE-conjugated annexin V in annexin V binding buffer (BD Biosciences; 0.01M HEPES, 140 mM NaCl, 25 mM CaCl_2_). After a further wash in annexin V binding buffer the cells were fixed using a BrdU staining kit (BD Biosciences). Nuclear DNA was digested with DNase I (Sigma) at 37°C for one hour prior to staining for BrdU epitopes. The stained cells were then analyzed by flow cytometry.

## Supporting Information

Figure S1 Assessment of cell death and proliferation in CD16^hi^, CD16^lo^ and LPR.FcRγ^−/−^ DN T cells. LPR.FcRγ^+/+^ (n = 5) and LPR.FcRγ^−/−^ mice (n = 5) were fed BrdU in the drinking water for 6 days, and then their splenocytes were stained for expression of TCRβ, CD16/32, CD4. CD8 and NK1.1 and with annexin V and analyzed by flow cytometry (same experiment as in Fig. 3B–C). **A.** BrdU and Annexin V staining for the other 8 mice not shown in Fig. 3B (n = 4 LPR.FcRγ^+/+^, top 2 rows showing the CD16hi and CD16lo subsets gated as shown in Fig. 3B; and n = 4 LPR.FcRγ^−/−^ mice, bottom row). Numbers inside plots reflect the percentages of gated cells falling into each quadrant. **B.** The percentage of live, proliferated (BrdU^+^ annexin V^−^) for all 10 mice is shown. One-way ANOVA p = NS.
(TIF)

Figure S2 Lack of evidence for other FcRγ-dependent regulatory cells in LPR mice. **A.** LPR.FcRγ^−/−^ mice aged 4 weeks received two injections of 4–5×10^6^ LPR.FcRγ^+/+^ (n = 3) or LPR.FcRγ^−/−^ (n = 5) NK cells, two weeks apart. After another two weeks, spleen and lymph node cell counts were determined. Two-way ANOVA p = NS for the effect of NK cell FcRγ expression. **B.** LPR.FcRγ^−/−^ mice aged 4 weeks received two cell injections of either LPR.FcRγ^+/+^ CD3^−^CD19^−^ cells (n = 3) or B6.LPR.FcRγ^−/−^ CD3^−^CD19^−^ cells (n = 3), 2 weeks apart. Cells derived from one-two donor mice (∼1–3×10^6^ per dose) were used for each injection, ensuring that an equivalent number of LPR.FcRγ^−/−^ and LPR.FcRγ^+/+^ cells were transferred on each occasion. After a further 4 weeks, total spleen and lymph node cell counts were determined. Unpaired t-test p = NS. **C.** Lymph node cells from LPR FcRγ^+/+^ and LPR FcRγ^−/−^ mice were intracellularly stained for FcRγ and Foxp3. Contour plots show Foxp3 and FcRγ expression within the CD4^+^ population. Results are representative of 9 mice per genotype.
(TIF)
