# Supplementary figures and images for: FcRγ Controls the Fas-Dependent Regulatory Function of Lymphoproliferative Double Negative T Cells
Source: PLoS One. 2013 Jun 6;8(6):e65253. doi: 10.1371/journal.pone.0065253 (PMC3675138; doi:10.1371/journal.pone.0065253)

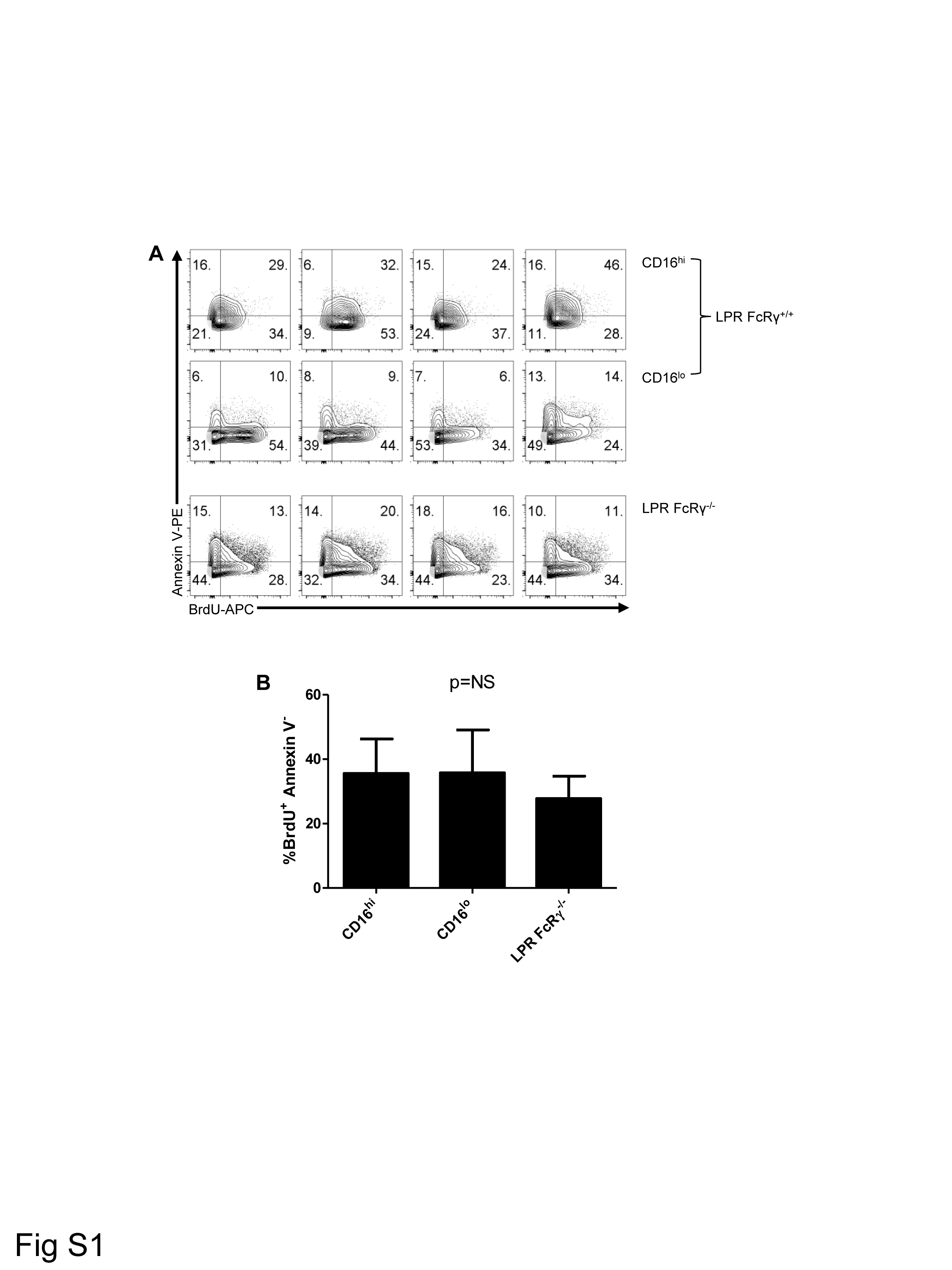

Supplement: Figure S1 — Assessment of cell death and proliferation in CD16hi, CD16lo and LPR.FcRγ−/− DN T cells. LPR.FcRγ+/+ (n = 5) and LPR.FcRγ−/− mice (n = 5) were fed BrdU in the drinking water for 6 days, and then their splenocytes were stained for expression of TCRβ, CD16/32, CD4. CD8 and NK1.1 and with annexin V and analyzed by flow cytometry (same experiment as in Fig. 3B–C). A. BrdU and Annexin V staining for the other 8 mice not shown in Fig. 3B (n = 4 LPR.FcRγ+/+, top 2 rows showing the CD16hi and CD16lo subsets gated as shown in Fig. 3B; and n = 4 LPR.FcRγ−/− mice, bottom row). Numbers inside plots reflect the percentages of gated cells falling into each quadrant. B. The percentage of live, proliferated (BrdU+ annexin V−) for all 10 mice is shown. One-way ANOVA p = NS. (TIF) [file pone.0065253.s001.tif]

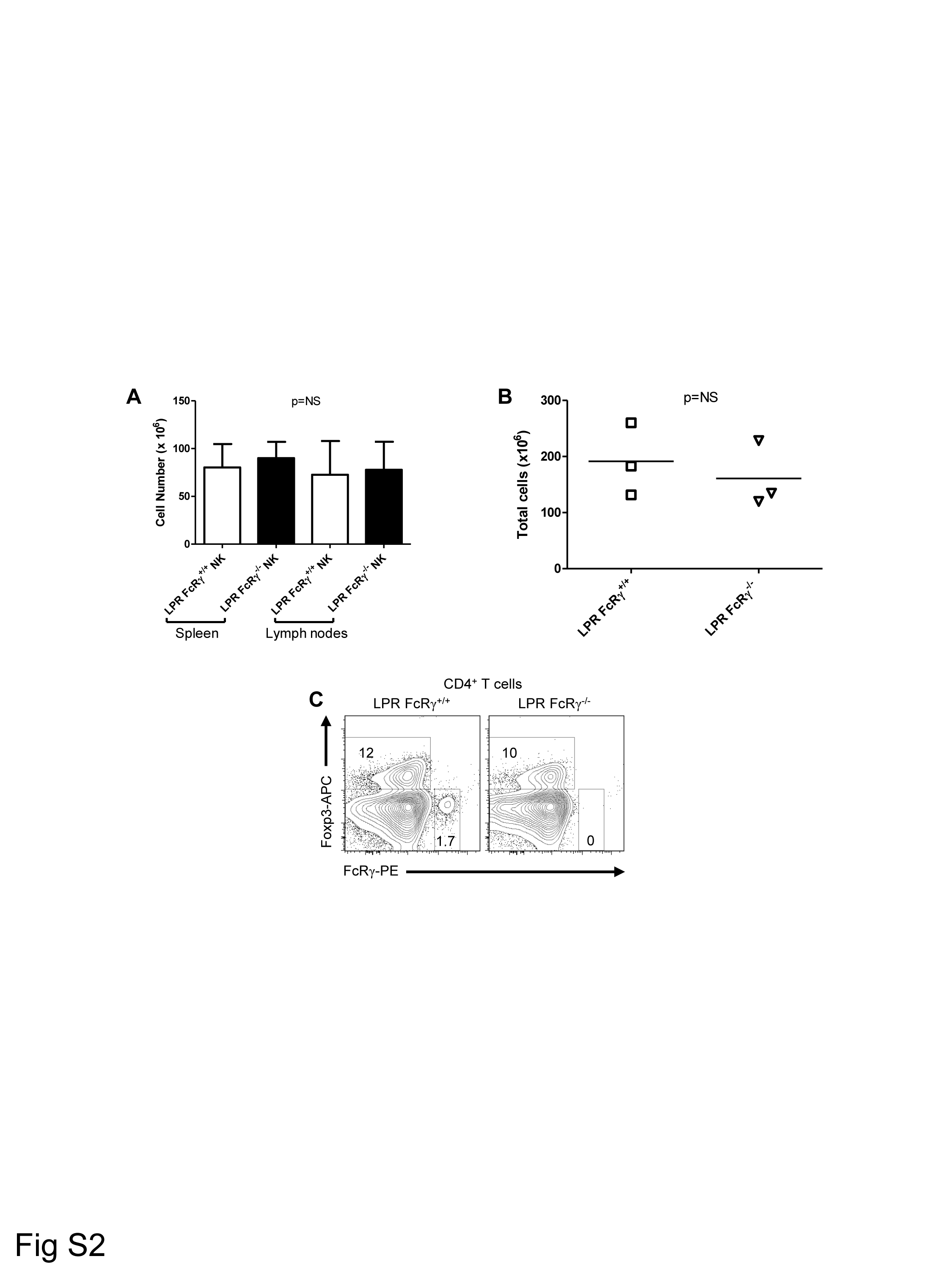

Supplement: Figure S2 — Lack of evidence for other FcRγ-dependent regulatory cells in LPR mice. A. LPR.FcRγ−/− mice aged 4 weeks received two injections of 4–5×10^6^ LPR.FcRγ+/+ (n = 3) or LPR.FcRγ−/− (n = 5) NK cells, two weeks apart. After another two weeks, spleen and lymph node cell counts were determined. Two-way ANOVA p = NS for the effect of NK cell FcRγ expression. B. LPR.FcRγ−/− mice aged 4 weeks received two cell injections of either LPR.FcRγ+/+ CD3−CD19− cells (n = 3) or B6.LPR.FcRγ−/− CD3−CD19− cells (n = 3), 2 weeks apart. Cells derived from one-two donor mice (∼1–3×10^6^ per dose) were used for each injection, ensuring that an equivalent number of LPR.FcRγ−/− and LPR.FcRγ+/+ cells were transferred on each occasion. After a further 4 weeks, total spleen and lymph node cell counts were determined. Unpaired t-test p = NS. C. Lymph node cells from LPR FcRγ+/+ and LPR FcRγ−/− mice were intracellularly stained for FcRγ and Foxp3. Contour plots show Foxp3 and FcRγ expression within the CD4+ population. Results are representative of 9 mice per genotype. (TIF) [file pone.0065253.s002.tif]
